# Online rumors during the COVID-19 pandemic: co-evolution of themes and emotions

**DOI:** 10.3389/fpubh.2024.1375731

**Published:** 2024-06-10

**Authors:** Chao Shen, Zhenyu Song, Pengyu He, Limin Liu, Zhenyu Xiong

**Affiliations:** School of Management, Nanjing University of Posts and Telecommunications, Nanjing, China

**Keywords:** public health emergencies, online rumors, theme evolution, emotional evolution, co-evolution

## Abstract

**Introduction:**

During public health emergencies, online rumors spread widely on social media, causing public information anxiety and emotional fluctuations. Analyzing the co-evolution patterns of online rumor themes and emotions is essential for implementing proactive and precise governance of online rumors during such events.

**Methods:**

Rumor texts from mainstream fact-checking platforms during the COVID-19 pandemic were collected and analyzed in phases based on the crisis lifecycle theory. The LDA topic model was applied to analyze the distribution of rumor themes at different stages. The Baidu AI Sentiment Analysis API was used to study the emotional tendencies of rumors at different stages. Line graphs were utilized to analyze the co-evolution characteristics of rumor themes and emotions.

**Results:**

During the COVID-19 pandemic, the themes of online rumors can be categorized into five types: epidemic prevention and control, panic-inducing, production and livelihood, virus dissemination, and social figures. These themes exhibited repetition and fluctuation at different stages of the pandemic. The emotions embedded in pandemic-related online rumors evolved with the progression of the pandemic. Panic-inducing rumors co-evolved with negative emotions, while epidemic prevention and control rumors co-evolved with positive emotions.

**Conclusion:**

The study results help to understand the public’s focus and emotional tendencies at different stages of the COVID-19 pandemic, thereby enabling targeted public opinion guidance and crisis management.

## 1 Introduction

Since the beginning of the 21st century, the occurrence rate of public health emergencies has significantly increased (1). Events such as SARS, avian influenza, and the Ebola outbreak have had profound impacts on global health and social progress (2). The COVID-19 pandemic, which emerged in December 2019, has had a wide-reaching impact, lasting for a prolonged period and resulting in a high mortality rate, causing global panic. As the virus spreads, online rumors also run rampant in cyberspace. These rumors pose a threat to society as serious as the public health emergency itself, jeopardizing social order and hindering information management efforts (3).

Research has found that social networking platforms have become breeding grounds for the spread of online rumors (4). While these platforms are crucial for real-time communication, they also serve as channels for the rapid dissemination of unverified claims and panic-inducing narratives. Captivating themes play a significant role in the spread of information through social media. Additionally, the inclusion of emotions in rumors significantly increases their likelihood of being shared, regardless of their veracity. This emotional packaging often resonates with people’s emotions, triggering chain reactions on social networks, further accelerating the spread and scope of rumors.

To effectively counter the spread of online rumors during public health emergencies, scholars have studied the characteristics of rumor diffusion during epidemics. Xiao et al. found that different rumor themes exhibit differentiated distribution patterns at different stages of the epidemic, necessitating targeted governance measures (5). Liu et al. found that emotional appeals contained in rumors significantly affect their survival time (6). It is evident that the spread of online rumors is closely intertwined with their textual content (7). In this context, our study primarily addresses the following three questions:

Question 1: What are the main themes of online rumors during the COVID-19 pandemic?

Question 2: What are the emotional tendencies contained in online rumor texts during the COVID-19 pandemic?

Question 3: How do the themes and emotions of online rumors co-evolve during the COVID-19 pandemic?

Co-evolution originally referred to the interaction patterns between two major organisms with close and evident ecological relationships (8), emphasizing the evolutionary relationship between interacting entities (9). In the context of online rumors, we believe that there is a mechanism of mutual interaction between the themes and emotions of online rumors as public health emergencies develop. We define the process of intertwining and influencing each other between the themes and emotions of online rumors as the co-evolution of themes and emotions.

In comparison with previous research, this study contributes in two main aspects. On the one hand, unlike previous studies that separately analyze the themes and emotions of rumor texts, this paper integrates both perspectives for analysis. On the other hand, by studying the co-evolution of themes and emotions of online rumors, this paper can reveal the underlying laws of rumor propagation during public health emergencies, aiming to provide insights for addressing online rumors during public health emergencies. Following the introduction, in Section 2, we review the relevant research on online rumors during public health emergencies, with a particular focus on their themes and emotions. Section 3 introduces the research design and framework, explaining the sources and roles of the research data. Section 4 elaborates on the analysis of themes and emotions of online rumors during the COVID-19 pandemic, and studies the co-evolution of the two. Section 5 presents the main findings of this study, along with its theoretical and practical significance, limitations, and future research directions. Finally, Section 6 provides a summary of the entire paper. To facilitate better comprehension of certain terms used throughout this article, we have provided a glossary (Table 1). Please refer to this glossary for enhanced understanding of the article’s content.

**Table 1:** Glossary.

Term	Definition
Baidu AI	Baidu AI refers to the artificial intelligence (AI) technology and platform developed by Baidu Inc. Baidu AI encompasses AI technologies and applications in various fields, including natural language processing, computer vision, speech recognition, intelligent recommendations, and more. The goal of Baidu AI is to provide more intelligent, personalized, and convenient services and products through AI technology to meet users’ needs in various scenarios.
Beijing Xinfadi Outbreak	The Beijing Xinfadi outbreak refers to the COVID-19 epidemic that erupted in the Xinfadi Agricultural Products Wholesale Market in Beijing in June 2020. The distinctive feature of this outbreak was the sudden appearance of new cases after 56 consecutive days without local new confirmed cases in Beijing, which sparked widespread attention and urgent responses. Following the emergence of the outbreak, the Beijing municipal government swiftly implemented a series of measures to control the spread of the virus, including market lockdowns, large-scale nucleic acid testing, and implementation of isolation measures.
China Internet Joint Rumor Refutation Platform	The China Internet Joint Rumor Refutation Platform, sponsored by the Cyberspace Administration of China (CAC)'s Illegal and Harmful Information Reporting Center and hosted by Xinhua News Agency, was launched on August 29, 2018. The platform relies on the National Network Rumor Refutation Mechanism, consisting of 104 units, to achieve collaborative discovery, verification, and refutation of rumor information. On this platform, rumor information will be clearly marked to remind the public to be vigilant and cautious.
Co-evolution	In this article, co-evolution refers to the mutual influence and interaction between the themes and emotions conveyed in rumors circulating during sudden public health emergencies. On one hand, the emotions embedded in rumors affect the public’s focus, thus making certain thematic rumors more likely to emerge and become hot topics. On the other hand, as thematic rumors emerge, resonance forms between the public’s emotions and the emotions conveyed in these themes, making the public more prone to believing and spreading such rumors. Consequently, the emotions embedded in rumors are further amplified.
Nanjing Outbreak	In July 2021, Nanjing experienced an outbreak of COVID-19. The outbreak originated at Lukou International Airport, where some staff tested positive during regular nucleic acid testing. Subsequently, the epidemic spread rapidly, leading to an increase in the risk level in multiple areas. The Nanjing municipal government promptly implemented a series of epidemic prevention and control measures, including the lockdown of high-risk areas, large-scale nucleic acid testing, and restrictions on population movement.
Rumor Filter	The Rumor Filter is an official debunking account launched by WeChat on October 17, 2014. WeChat collaborates with various partners such as People’s Daily Online, Guokr.com, and Dingxiang Garden to jointly filter rumors on the WeChat platform through content operation and rumor identification. WeChat aims to enhance netizens’ ability to discern the truth by providing authoritative interpretations and filtering out online rumors with facts.
Shijiazhuang, Hebei Outbreak	In early January 2021, Shijiazhuang city reported multiple confirmed cases and asymptomatic carriers. Within just 3 days, Hebei Province reported 59 patients, including 14 confirmed cases and 40 asymptomatic carriers in Shijiazhuang city. Shijiazhuang promptly entered a “wartime state,” announcing city lockdowns and initiating city-wide nucleic acid testing. Meanwhile, the Education Bureau issued an urgent notice, deciding to suspend offline education and teaching activities in all primary and secondary schools and kindergartens in the city.
Tencent Fact Check	Tencent Fact Check is a new program launched under the Tencent News channel, dedicated to creating a platform for public news verification. Its goal is to verify and swiftly counter various types of false news, lacking news, rumors, phishing posts, and marketing posts. It aims to trace the origins and investigate the truth behind messages that people are interested in but lack context.

## 2 Related works

### 2.1 Research on online rumors of public health emergencies

Public health emergencies are deeply intertwined with public health and safety, naturally drawing public attention and shaping discourse. This phenomenon creates an “information cocoon” that significantly impacts group cognition and behavior through diverse channels, including news, notifications, and rumors (10). Research into rumors during public health emergencies in China gained prominence during the SARS outbreak (11) and experienced resurgence during the COVID-19 pandemic. Scholars have examined the shared characteristics of online rumors during public health crises, including rapid dissemination, information overload, and substantial adverse effects (12). They argue that differences in technological environments, event elements, and public perceptions lead to notable distinctions in rumors during different public health emergencies (13).

The key focus of research on online rumors during public health emergencies revolves around several themes (14), including differences in sources, the dissemination of rumors on various topics (15), and how these themes evolve over time. Internet rumors have morphed in form, content, and representation in the post-pandemic era, often taking on variants that diverge from mainstream ideologies and significantly impacting the establishment of government discourse mechanisms. Scholars have delved into three aspects of online rumors: topic prominence, topic content, and topic associations, with topic prominence reflecting the focal points of societal attention (16). Theme content represents the semantic dimension of online rumor research.

Drawing from a comprehensive dataset of users collected from the Weibo platform during the 2020 COVID-19 pandemic, Liu developed an interactive infection model to explore the dynamics of multiple rumor participants within different intervention contexts, yielding varied outcomes concerning psychosocial reactions, resistance, and exhaustion periods (17). Guo et al. utilized COVID-19 as a case study to investigate rumor propagation from the perspective of information access. Their study demonstrated that information obtained from social media inhibits rumor dissemination, with rumor beliefs acting as mediators. Information obtained from traditional media diminishes the influence of information acquired from social media on rumor beliefs, and critical thinking mitigates the positive effects of rumor beliefs on rumor sharing (18).

COVID-19, as a major public health emergency since the establishment of the People’s Republic of China, has garnered significant attention due to its rapid virus mutations, extensive transmission, and prolonged response period. This has made the evolution of the themes, content, and intensity of public health emergency narratives a central research focus. Xiao et al. conducted an analysis exploring the dynamic features of internet rumors during the COVID-19 pandemic. They considered the pandemic’s progression as a time series, providing a systematic and objective assessment of temporal shifts in internet rumor topics and the notable changes in public sentiment within this unique and intensively generated context (5). Yan studied the evolution of online rumors throughout the complete cycle of COVID-19, basing his analysis on the development and prevalence of public opinion. His conclusion is that while the themes of rumors may shift focus at different stages, they also exhibit cyclic fluctuations. Simultaneously, public emotions frequently oscillate. Additionally, the same rumors often resurface (19).

In summary, while most studies on the themes of online rumors during public health emergencies have concentrated on macroscopic content mechanisms, thematic intensity, and thematic associations, there has been a lack of attention to the thematic evolutionary characteristics of rumors and rumor-refuting information. These studies have overlooked the spatial and temporal similarities and differences at each stage of the life cycle. In terms of research methods, content analysis, textual analysis, and case studies have predominated, with limited exploration of the sudden variability and differentiation of rumor themes during public health emergencies on rumor-refuting platforms, particularly within a time-series context.

### 2.2 Research on the evolution of online rumor themes and sentiment analysis

Public health emergencies, often characterized by the proliferation and mutation of viruses, exhibit persistent and evolving traits. These dynamics give rise to derivative and recurring themes in online rumors that correspond to the progression of events, subsequently triggering fluctuations in public sentiment. Scholars have conducted extensive research on the evolution of topics and sentiment analysis within online rumors. They have introduced dynamic topic models (DTM) that incorporate topic time slicing, building upon the foundation of the Latent Dirichlet Allocation (LDA) model that considers document-topic probability distribution (20).

Subsequent researchers have further advanced this field by introducing models such as the Discrete Dynamic Topic Model (DDTM) (21) and the Continuous Dynamic Topic Model (CDTM) (22), among others, which have found application in the study of public health emergencies. Deng et al. proposed an interactive topic modeling approach to extract the public’s information needs from social media and track their evolution. They conducted retrospective analyses, using the Tianjin Port explosion incident as an example, to discover and visualize evolving topics (23). Cinelli et al. conducted a comparative analysis of user activity across five different social media platforms during the COVID-19 health emergency. They used an epidemic model to fit information dissemination to the underlying reproduction numbers of each platform. Their findings underscored the significance of the time frame as a benchmark for studying the dynamics of content consumption before and after critical events, especially when the accuracy of information is at risk. Importantly, they noted that reliable and questionable sources of information did not exhibit different propagation patterns (24).

Furthermore, Garcia et al. leveraged Twitter data to calculate and analyze the content of hot topics and their associated sentiments among both US and Brazilian internet users. This study aimed to explore the reasons for the varying content and sentiments across different topics (25). Yang et al. developed a stage model to analyze the spatial and temporal distribution of public panic during public health emergencies, providing practical insights (26). An et al. explored differences in the topics followed by Weibo and Twitter users during public health emergencies, considering language and user nationality, revealing patterns of topic evolution over time (27).

Although existing research has elucidated many characteristics of online rumors during public health emergencies, including themes and emotions, their focus on co-evolution is limited. Some scholars have explored the influence of emotions embedded in online rumor texts on their spread, but there has been no associated research with themes (28).

In summary, existing research has largely focused on the thematic changes of online rumors during public health emergencies and the fluctuation of public emotions. Regarding thematic analysis, the emphasis has been on theme identification and popularity analysis, while emotional research has centered on sentiment measurement and evolution. However, these studies often examine themes or emotions in isolation, failing to reveal the co-evolutionary patterns of themes and emotions.

## 3 Research design

### 3.1 Research ideas

**Figure 1:**
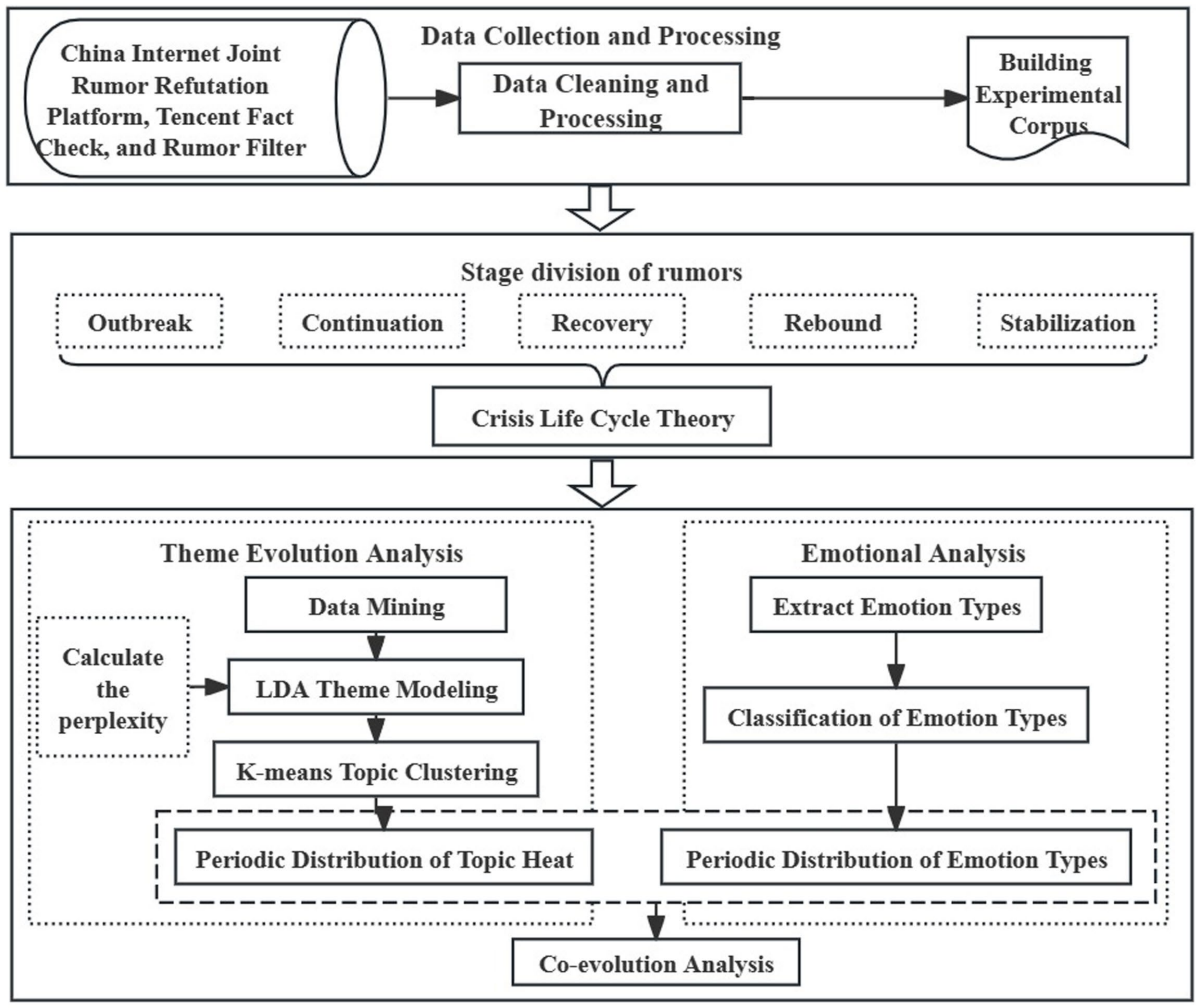
Research framework.

Managing public health emergencies is a multifaceted endeavor. On one hand, it entails mobilizing a wide array of social resources, including medical facilities, personnel, and supplies, to facilitate offline prevention and control efforts. On the other hand, it involves addressing diverse online public opinions, guiding emotions, and disseminating prevention and control knowledge to cultivate positive expectations. This approach aims to achieve integrated offline and online prevention and control strategies. During public health emergencies, a minority of internet users spread rumors through social media platforms for attention, traffic, or to voice personal grievances. These rumors, widely disseminated, can disrupt government policies and even incite collective confrontation. Although legislation criminalizes the fabrication and dissemination of online rumors, governing them remains challenging due to covert dissemination and difficulty in obtaining evidence. To tackle this, China has adopted a crowdsourcing governance model (29), establishing platforms such as the China Internet Joint Rumor Refutation Platform,^1^ Rumor Filter,^2^ Tencent Fact Check,^3^ etc., to assist the public in identifying and addressing online rumors. This article examines COVID-19 pandemic online rumors, studying their evolution from thematic and emotional perspectives. Utilizing crisis lifecycle theory (30) to analyze thematic cycles and investigating the co-evolution of thematic and emotional aspects at different event stages, the study aims to illuminate the governance of public health emergency online rumors, as illustrated in Figure 1.

### 3.2 Data collection and processing

We selected the China Internet Joint Rumor Refutation Platform, Tencent Fact Check, and Rumor Filter as our data sources (5, 31). These three platforms are highly credible and authoritative in the field of online public opinion supervision and rumor refutation in China. These platforms typically employ a multi-faceted verification approach, engaging with authoritative agencies, experts, scholars, and relevant stakeholders to verify the authenticity of information, ensuring that it qualifies as a rumor. Simultaneously, platforms track and trace the dissemination pathways of information. By understanding the channels and routes through which information spreads, they can better identify the origins of rumors. Using Python, we collected text data explicitly marked as rumors from these platforms and saved them in an Excel file, spanning from January 1, 2020, to November 30, 2021, totaling 1,584 entries. We manually verified the collected data. Firstly, due to the presence of duplicate sources across the three platforms, there were instances of repeated rumor texts, such as “Taking vitamin C effervescent tablets can prevent the novel coronavirus,” which appeared on all three platforms. We retained one instance of such data and removed the rest to enhance the quality of the dataset. Secondly, although most rumors during the data collection period revolved around the COVID-19 pandemic, there were also rumors unrelated to the pandemic. For example, “Eating strawberries can cause hemorrhagic fever.” We excluded such data. After these steps, we retained a total of 1,249 valid data entries. We provide three examples of the data format (Table 2) to illustrate the structure of our dataset.

**Table 2:** Data examples.

Time	Rumor content	Source
2020-01-18	Unexplained pneumonia in Wuhan is due to SARS virus.	Tencent Fact Check
2020-06-16	Several tens of thousands of people from the Xinfadi Market in Beijing have been transported to quarantine in Tangshan, Hebei Province.	China Internet Joint Rumor Refutation Platform
2021-01-06	Academician Zhong Nanshan arrives in Shijiazhuang to combat the epidemic.	Rumor Filter

To understand the thematic distribution of online rumors during the COVID-19 pandemic, we employed the LDA topic model for topic identification. Latent Dirichlet Allocation (LDA) is a text mining tool that assumes documents are composed of latent topics, which are in turn defined by distributions over vocabulary. In the LDA model, each document is viewed as a mixture of topics, each with a specific vocabulary distribution. Each word in a document is generated through a random process where a topic is first selected and then a word is chosen from the distribution of that topic.

Mathematically, the LDA model formalizes this process using the multinomial distribution and the Dirichlet distribution. The probability of generating a word (*w*) in a document (*d*) is represented as the weighted sum of probabilities over all possible topics (*z*):


$$P\left(w|d\right)=\sum_{k=1}^{K}P\left(w|z_{k}\right)P\left(z_{k}|d\right)$$

In the equation, $P\left(w|z_{k}\right)$ represents the probability of generating a word (*w*) under topic (*k*), and $P\left(z_{k}|d\right)$ represents the probability of the document (*d*) containing topic (*k*).

Furthermore, the distributions of topics in documents and words in topics are both assumed to follow the Dirichlet distribution. For instance, the Dirichlet distribution for the topic distribution *θ* of a document can be expressed as:

$$P\left(\theta \left|\alpha \right.\right)=\frac{\Gamma \left(\sum_{i=1}^{K}\alpha_{i}\right)}{\prod_{i=1}^{K}\Gamma \left(\alpha_{i}\right)}\prod_{i=1}^{K}\theta_{i}^{\alpha_{i}-1}$$

where *θ* is the topic distribution vector, and *α* is the parameter vector of the Dirichlet distribution (32–34).

We preprocess the text data by segmenting it into words, removing stopwords, and conducting other data preprocessing operations. We use perplexity as the evaluation metric to determine the optimal number of topics (5). Perplexity is a metric used to measure the quality of a probabilistic model’s predictions for samples, commonly applied in language models and topic models within natural language processing. Rooted in information theory, it serves as a means to compare predictive capabilities among different models. Lower perplexity indicates better predictive power, suggesting reduced uncertainty in the model’s understanding of the data. In topic models such as LDA, perplexity is commonly employed to evaluate the clustering quality of the model over a document collection. The calculation formula is as follows:

$$\mathrm{Perplexity}\left(D\right)=\exp\left(-\frac{\sum_{d=1}^{M}\log P\left(w_{d}\right)}{\sum_{d=1}^{M}N_{d}}\right)$$

where *D* is the document collection, *M* is the number of documents, $w_{d}$ is the word in document *d*, $N_{d}$ is the length of document *d*, and $P\left(w_{d}\right)$ is the probability of the word in document *d*.

**Figure 2:**
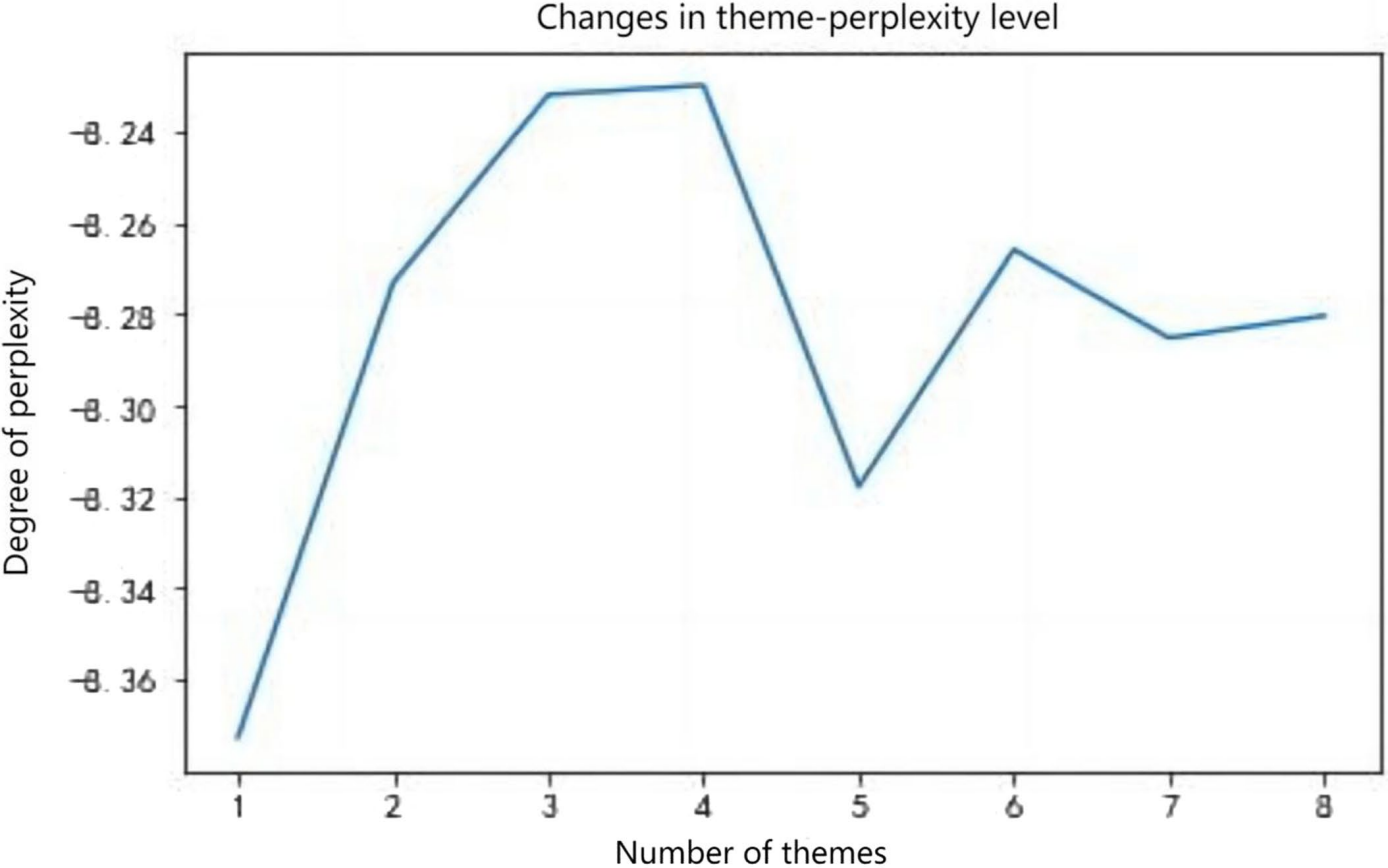
Calculation of perplexity.

Experimental results show that when the number of topics is set to 5, the model achieves the lowest perplexity. Furthermore, as the number of topics continues to increase, the decrease in perplexity is relatively small. Therefore, we determine the optimal number of topics to be 5 (Figure 2). Below are the five categories of rumor topics:

1. Epidemic prevention and control (keywords include confirmed cases, epidemic, lockdown).
2. Panic-inducing (keywords include red alert, new cases, positive tests).
3. Production and livelihood (keywords include supermarket, supplies, masks).
4. Virus dissemination (keywords include vaccine, Isatis indigotica, alcohol).
5. Social figures (keywords include Zhong Nanshan, media, reporting).

Based on this, we applied the K-means algorithm for topic identification (35, 36). The K-means clustering algorithm is a classic unsupervised learning method. Its aim is to partition the samples in a dataset into a pre-specified number of clusters, such that samples within the same cluster are highly similar, while samples across different clusters are dissimilar. The core idea of K-means clustering is to optimize the compactness within clusters through an iterative process, namely minimizing the distance between samples and cluster centroids. The basic steps of the K-means clustering algorithm are as follows:

1. Initialization: Randomly select K samples as the initial cluster centers.
2. Assignment: Assign each sample to the cluster whose center is nearest.
3. Update: Recalculate the center of each cluster, typically by taking the mean of all samples in the cluster.
4. Iteration: Repeat the assignment and update steps until a stopping condition is met, such as when the change in cluster centers is below a certain threshold or after a predetermined number of iterations.

The objective function of the algorithm is to minimize the sum of squared errors (SSE) within clusters, expressed as:


$$SSE=\sum_{i=1}^{k}\sum_{x\in C_{i}}||x-\mu_{i}{\left|\right|}^{2}$$

where *k* represents the number of clusters, *C_i_* represents the set of samples in the *i*-th cluster, *x* represents a sample point in the cluster, *μ_i_* represents the centroid of the *i*-th cluster, and $x-\mu_{i}$ represents the Euclidean distance between the sample point *x* and the centroid *μ_i_*.

The final results, as shown in Table 3, indicate a relatively high proportion of rumors in the categories of epidemic prevention and panic-inducing. These categories require particular attention.

**Table 3:** Classification of online rumor themes.

Type	Definition	Example	Quantity	Proportion (%)
Epidemic prevention and control	Rumors regarding the current status and future trends of the COVID-19 pandemic, including rumors about pandemic treatment, new area controls, and local policies.	Hangzhou lockdown for novel pneumonia prevention and control.	418	33.47%
Panic-inducing	Rumors fabricated, exaggerated, or manipulated by internet users, organizations, etc., with the intention of causing panic and social crisis during public health emergency.	The armed police will take over Shanghai’s communities.	497	39.79%
Production and livelihood	Rumors about abnormal operations of regional institutions, disruptions in daily life such as hoarding of epidemic prevention supplies, and financial fraud.	Nanjing will implement a three-day silence citywide starting tomorrow.	127	10.17%
Virus dissemination	Rumors concerning virus prevention, drug treatment, epidemic disinfection, and control measures.	Are Delta and other variants spreading in Beijing?	162	12.97%
Social figures	Rumors related to public figures such as medical experts, government representatives, and research scholars.	A junior high school student dies from severe COVID-19.	45	3.60%

## 4 Results

The outbreak of the novel coronavirus exhibits certain fluctuations, primarily stemming from localized outbreaks that occur within specific time periods. This paper, based on the Crisis Lifecycle Theory (30, 37), identifies three key nodes in the timeline: the June 2020 outbreak at Beijing Xinfadi, the January 2021 outbreak in Shijiazhuang, Hebei Province, and the July 2021 outbreak in Nanjing, Jiangsu Province, to divide the lifecycle of online rumors. During the pandemic, these three regions experienced large-scale confirmed cases, representing typical regional outbreaks. The events garnered significant attention during the current timeframe. The period from January to March 2020 is designated as the outbreak phase, June to August 2020 as the continuation phase, January to March 2021 as the recovery phase, July to August 2021 as the rebound phase, and September to November 2021 as the stabilization phase (following the conclusion of the Nanjing outbreak and a decrease in event intensity). Subsequent analyses will be conducted based on these defined stages.

### 4.1 The migration of online rumor themes in public health emergency

Theme content and popularity are two crucial dimensions in the study of online rumors, reflecting the focal points and trends of public attention during public health emergencies. During such events, the themes and popularity of online rumors exhibit dynamic and uncertain characteristics, often manifesting as repetitive derivations. Leveraging Dynamic Topic Modeling (DTM) (20) to obtain sequential models of theme evolution in organized document corpora, we comprehensively analyze the mechanisms driving the evolution of themes in online rumors during public health emergencies. Since rumor popularity gradually dissipates during the stabilization phase, we do not study the characteristics of rumors during this period.

**Figure 3:**
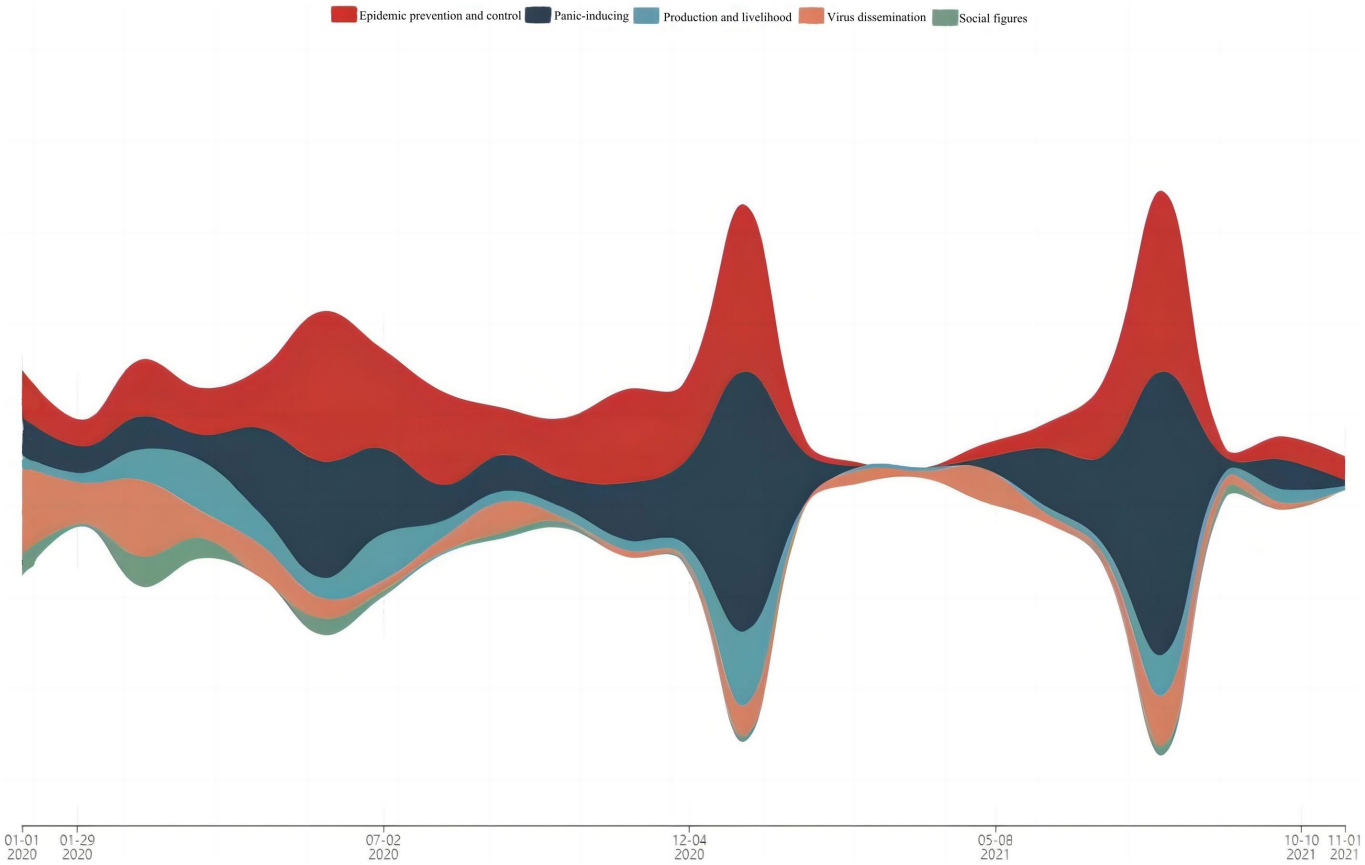
Evolutionary river map of rumor themes.

The Dynamic Topic Model (DTM) is an extension of Latent Dirichlet Allocation (LDA), a generative model used to analyze the evolution of topics over time in a collection of documents. Specifically, DTM divides the text into multiple time slices and assumes that each document within a time slice follows the same-dimensional topic model. However, the topic distributions and word distributions in each time slice vary over time. DTM predicts the topic and word distributions in the next time slice based on those in the previous time slice, thus forming a dynamic evolution of topics (20). We analyzed the evolutionary patterns and dissemination intensity of rumors across the five thematic categories, employing ThemeRiver to visualize the evolutionary trends of different thematic rumors (Figure 3). The width of the river indicates the dissemination intensity and duration of the rumors, while also providing insights into the priority of evolution for the five thematic categories over the course of the epidemic timeline. Additionally, based on the delineated epidemic stages, we conducted statistical analyses of the thematic popularity of online rumors (Table 4).

**Table 4:** Ranking of public health emergency rumor popularity.

Event stage	Epidemic prevention and control	Creating panic	Production and life	Virus spreading	Social figures
Outbreak	Relatively high	Medium	Low	high	Relatively high
Continuation	High	Relatively high	Medium	Relatively high	Low
Recovery	Relatively high	High	Medium	Relatively high	Low
Rebound	Relatively high	High	Relatively high	Medium	Low

Table 4 presents the popularity of online rumors across different thematic categories at various stages during the study period of the public health emergency. It can be observed that during the outbreak phase of major infectious disease rumors, rumors related to virus dissemination and epidemic prevention and control themes exhibit strong vitality. Public attention focuses primarily on the virus itself, including topics such as “possible virus origins,” “modes of virus transmission,” “virus virulence,” and “social distancing.” The characteristics of virus transmission sustain public interest in information regarding individual isolation and regional containment measures to disrupt virus transmission channels. Some derivative topics revolve around “virus prevention,” “drug treatment,” and “physical interventions.”

During the continuation phase, rumor themes concentrate on epidemic prevention and control and panic-inducing categories, with public attention shifting toward topics like “expressing personal emotions,” “impacts on travel/life,” “duration/extent of containment measures,” as well as derivative rumors concerning “vaccine development information,” “progress of overseas outbreaks,” and “epidemic tracing situations.” Given the unknowns surrounding the pathology, transmission routes, preventive measures, and treatment options for infectious diseases, coupled with the disruptive impact of epidemic prevention and control measures on normal life, feelings of anxiety, panic, and helplessness repeatedly surface among individuals. Consequently, internet users, driven by a desire for verification and concern, disseminate unconfirmed information on public health emergencies, further fueling the rise in popularity of panic-inducing rumors.

During the recovery phase, panic-inducing and epidemic prevention and control rumors dominate public discourse, with rumor themes focusing on topics such as “fabricated diagnoses/positives/virus mutations,” “drug reserves,” and “herd immunity,” as well as region-specific rumors involving “missionaries” and “conspiracy theories.”

In the rebound phase, public attention shifts toward a myriad of information regarding “virus transmission sources,” “epidemic spread,” and “international epidemic impacts.” Some rumors are associated with outbreak regions, with airport-related epidemic transmissions giving rise to rumors such as “1,000 new cases confirmed in Nanjing” and “Nanjing lockdown,” spreading widely across the internet. These sporadic outbreaks exacerbate public anxiety, while panic-inducing rumors exaggerate the severity of the epidemic, intensifying the spread of rumors.

Throughout the different stages of the public health emergency, there are significant differences in the popularity of rumor themes. During the outbreak phase, public attention focuses on virus transmission pathways, scope, and the epidemic itself. As the epidemic spreads and medical resources become strained, public attention shifts to vaccine development, isolation measures, treatment options, and individual impacts. During the continuation phase of the epidemic, public discourse transitions to vaccine efficacy, overseas imports, and blocking transmission sources, reflecting concerns about societal impacts. With the repeated fluctuations of the epidemic, rumors related to epidemic prevention and control and panic-inducing themes become recurring hot topics for the public, constituting the norm for rumors during public health emergencies. Rumors related to production and livelihood consistently maintain low popularity. From the perspective of theme migration, epidemic prevention and control rumors consistently maintain high popularity, while virus dissemination rumors are concentrated in the outbreak phase of the epidemic. Panic-inducing rumors spread widely immediately after the outbreak of the event, followed by the emergence of production and livelihood rumors and social figures rumors. The diversity and fluctuation of online rumor themes during public health emergencies reflect changes in public awareness and attention toward the event itself. The emotional characteristics of rumor themes resonate with internet users, serving as important reasons for the derivation and recurrence of rumors. Further analysis of the emotional characteristics of online rumors is necessary.

### 4.2 The emotional evolution of online rumors in public health emergency

The sentiment of online rumors reflects the inclination of internet users toward events, making it a significant factor influencing the spread of rumors. Negative rumors tend to evoke extreme emotions among internet users, while positive rumors embody the public’s optimistic outlook for the future (38). Negative rumors exhibit a functional characteristic, implying their negative intent, containing imprecise negative information directed toward individuals, society, or the nation (39). Neutral rumors emphasize adopting a neutral stance toward rumors, aiming to present standardized statements, plain text, and complete characterizations (40). The sentiment of online rumors fluctuates across different stages of event development. To investigate the emotional tendencies contained within online rumor texts during the pandemic, we utilized the Baidu AI sentiment analysis tool for sentiment polarity recognition. The division results of emotional tendencies are shown in Table 5, where 0, 1, and 2 correspond to negative sentiment, neutral sentiment, and positive sentiment, respectively.

**Table 5:** Example of extracting emotion from online rumors in public health emergency.

Rumor text	Entity word extraction	Confidence	Negative probability	Positive probability	Emotional tendencies
Ruili City, a man surnamed Jin jumped from the 4th floor of a hot spring hotel to escape, by the emergency rescue and timely hospital treatment, the current vital signs stable	Ruili, Hot Spring Hotel, Jumping from a building, suicide, Emergency, Rescue, Hospital, Cure, Life, Physical sign, stable	0.948	0.976	0.023	0
Vaccination can receive 120 yuan red packet and 30 yuan car allowance, introduce a person to play can also get 20 yuan referral fee, next month to start mandatory play still no red packet, the opportunity is rare	Vaccination, 120RMB, Red Packet, Car allowance, Referral Fee, Next month, Forced, Opportunity	0.993	0.002	0.997	2
Fujian Xiamen Third Hospital nucleic acid test more than 200 positive is a rumor	Fujian, Xiamen Third Hospital, Nucleic acid testing, Positive	0.567	0.194	0.805	1

Examining the sentiment tendencies of the sample data, we found that 48.8% of rumors were negative, 45% were positive, and 6.2% were neutral. Notably, negative and positive sentiment rumors exhibited similar proportions, while neutral rumors were concentrated primarily within the social figure and production and life categories. To track the changes in rumor sentiment throughout the epidemic, we created a table illustrating the evolution of sentiment tendencies (Table 6).

**Table 6:** Percentage of emotional tendency of rumors in each stage.

Life cycle	Outbreak	Continuation	Recovery	Rebound
Positive emotions	47.31%	55.84%	43.30%	38.97%
Negative emotions	43.71%	39.61%	52.92%	58.01%
Neutral emotions	8.98%	4.55%	3.78%	3.02%

The evolution of sentiment in online rumors during public health emergencies exhibits distinct characteristics: a rise and fall in the heat of positive rumors, a decline followed by an increase in the heat of negative rumors, and relatively low heat for neutral rumors. Positive and negative rumors maintain high heat levels across all stages, while neutral sentiment rumors have a lower proportion, indicating that public emotions are a primary driving force behind rumor dissemination. During the outbreak period, positive rumors show high heat levels as the event’s status and public perceptions remain uncertain, with positive rumors reflecting public expectations. In the sustained period, the heat of positive rumors gradually rises, while the heat of negative rumors decreases. The implementation of relevant control measures and timely information dissemination alleviate public anxiety, resulting in the peak heat of positive rumors. In the recovery and rebound periods, accumulating negative emotions among the public due to the event’s recurrence and unmet expectations lead to lower heat levels for positive sentiment rumors and sustained rebounds in negative sentiment rumors. As the event evolves, the sentiment of online rumors exhibits significant fluctuations, and analyzing its synergistic relationship with themes can explain the complexity of rumor dissemination.

### 4.3 The synergistic mechanisms of online rumor themes and emotions

**Figure 4:**
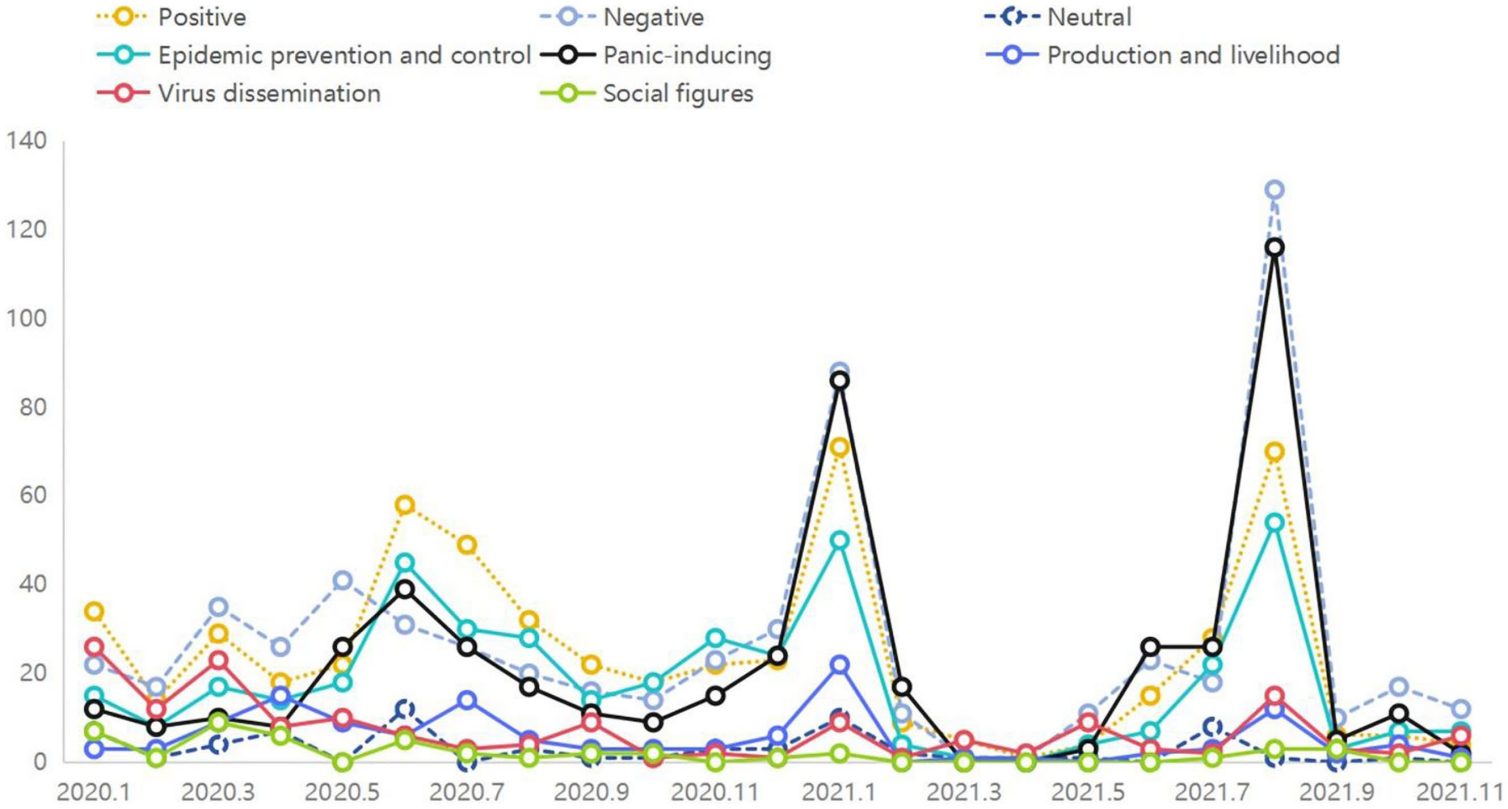
The trend of thematic shifts and emotional changes in online rumors.

The themes of online rumors evolve dynamically with the development of the epidemic, while the sentiment of online rumors reflects the psychological changes of netizens during the epidemic, manifested as collective behavior. The derivativeness, repetitiveness, and polarization, dissolution, and fluctuation of theme emotions in online rumors collectively drive the dissemination and evolution of online rumors, making them highly resilient during public health emergencies. Studying their co-evolution is fundamental to governing online rumors. This study employs line graphs to illustrate the co-evolution mechanism between epidemic-related online rumor themes and sentiments, utilizing five categories of rumor themes and three categories of emotional tendencies for analysis (Figure 4).

In the outbreak period, spanning from January to March 2020, the spread of online rumors was predominantly characterized by negative sentiments. This was due to the severe harm inflicted on public health, leading to public worries and panic. The lack of understanding intensified psychological anxiety, prompting individuals to seek information and verification. Rumors related to virus spread, epidemic control, and panic induction spread rapidly during this phase.

During the sustained period, from June to August 2020, there was a notable increase in rumors with positive sentiments. Rumors related to epidemic control showed an increasing trend in popularity, as effective implementation of control measures reduced the spread of the virus and alleviated public concerns. Negative rumors gradually decreased, similar to the changes observed in panic-inducing rumors. Rumors regarding social resumption and virus transmission also saw a decline in popularity. It is evident that rumors concerning epidemic control spread along with positive sentiments, reflecting public anticipation for a continued improvement in the epidemic situation. Conversely, rumors inducing panic spread along with negative sentiments, showing a decreasing trend in popularity.

During the recovery period, from January to March 2021, data indicated a simultaneous increase and decrease in the popularity of negative and panic-inducing rumors, respectively. Considering the characteristics of the epidemic during this phase, with the resurgence of the epidemic, rumors combined regional characteristics and virus origin information, reflecting public negative sentiments. Simultaneously, the popularity of positive rumors reached its peak. Rumors related to epidemic control and production and life showed an initial increase followed by a decrease, reflecting positive tendencies.

In the rebound period, from July to August 2021, data characteristics indicated that the popularity of negative rumors reached a third peak, and panic-inducing rumors exhibited a similar trend. Positive rumors slightly decreased compared to the previous phase, with changes in epidemic control rumors mirroring the magnitude of the changes. During this phase, panic-inducing and virus-spreading rumors reflected public fears and concerns. The popularity of these two types of rumors continued to increase, with negative sentiments gradually accumulating and experiencing significant fluctuations within a short period.

In the stable period, from September to November 2021, the number of rumors decreased rapidly, indirectly reflecting significant achievements in epidemic control by the government. Panic-inducing rumors no longer spread widely, and the trend of epidemic control rumors stabilized. Overall, the emotions conveyed by rumors remained negative during this phase, with negative sentiments gradually dissipating, and the positive sentiment curve steadily declining. During this phase, various types of rumors gradually faded from public view, and netizens’ emotions were promptly calmed. Individuals became more concerned about the virus situation due to uncertainties about the future development of the epidemic and the impact of the rebound period’s panic, resulting in the expression of predominantly negative sentiments in rumor content.

**Figure 5:**
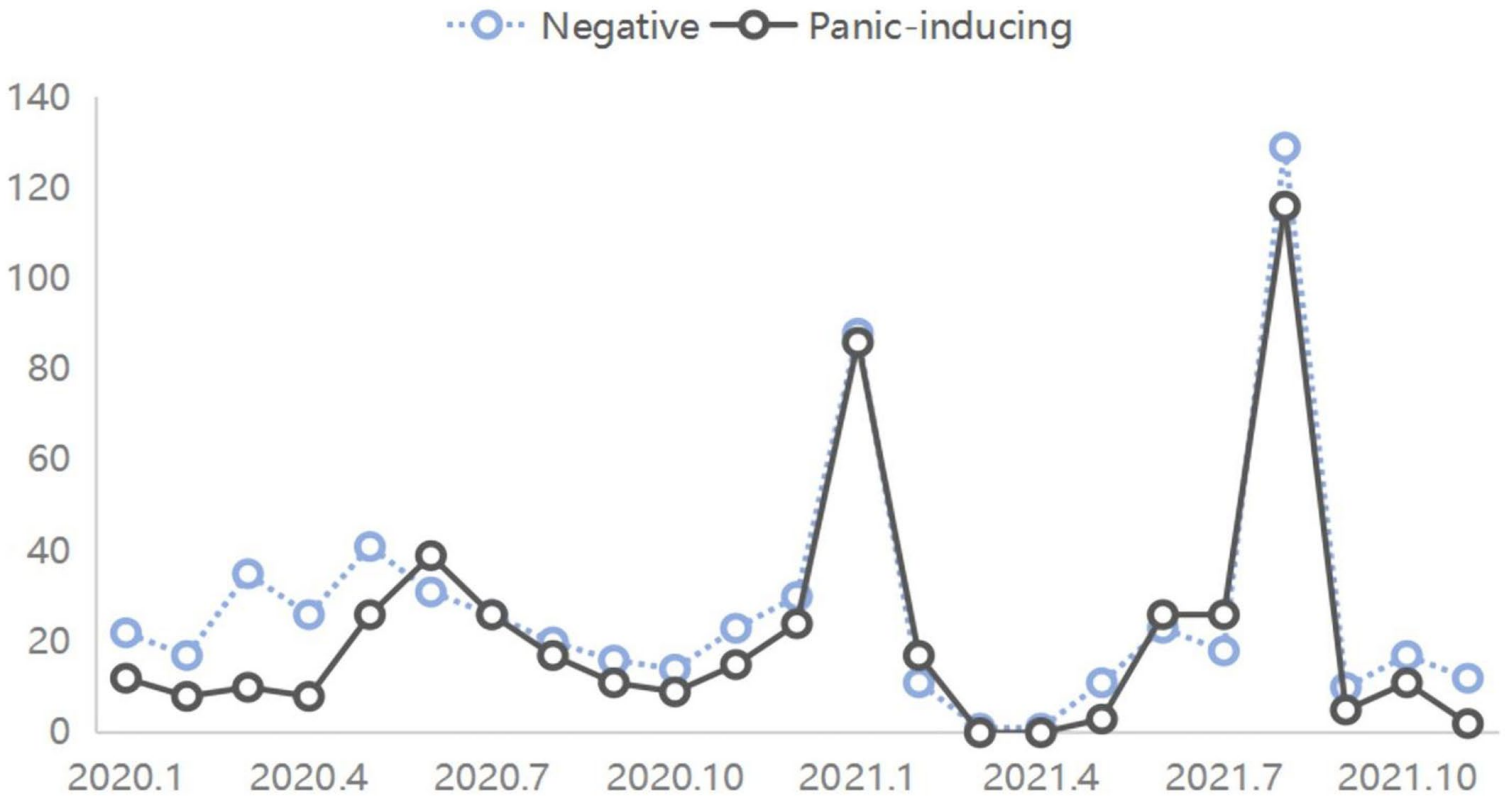
The co-evolution of panic-inducing rumors and negative emotion.

**Figure 6:**
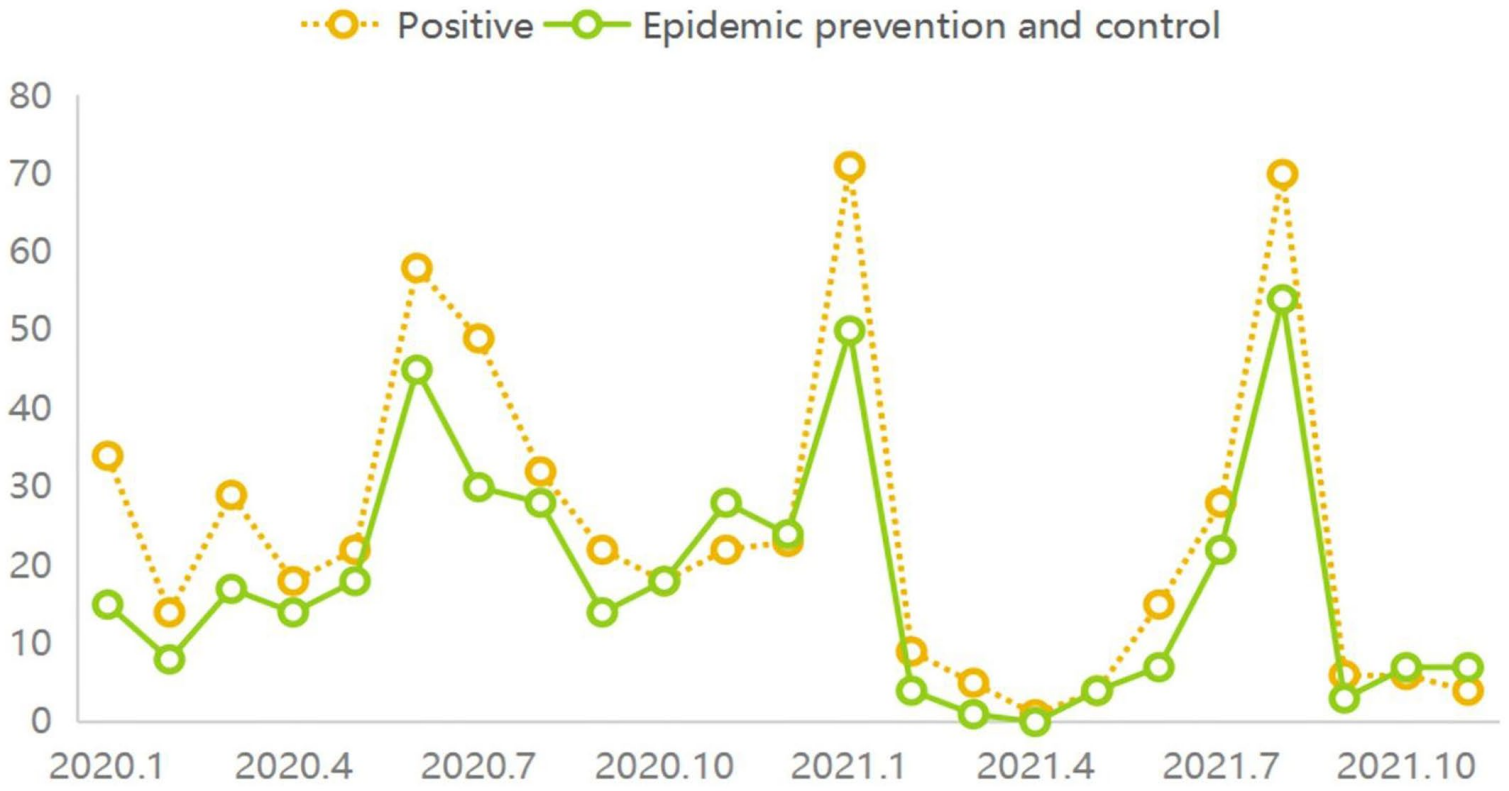
The co-evolution of epidemic prevention and control rumors and positive emotion.

Further analysis of the curves revealed a co-evolution characteristic between panic-inducing rumors and negative sentiments (Figure 5) and between epidemic control rumors and positive sentiments (Figure 6). In the early stages of the epidemic, the sudden outbreak instilled fear among the public, leading to the proliferation of various negative discussions on social media platforms. As disparate topics gradually developed into fixed themes with the progression of the epidemic, panic-inducing rumors emerged as a distinct category. These rumors triggered worries and fears among netizens, making them susceptible to believing and disseminating rumor content. In such circumstances, panic-inducing rumors further propagated, amplifying the negative sentiments contained in the rumor content. Conversely, as the epidemic unfolded, various regions implemented robust emergency measures. The proactive implementation of control measures brought a ray of hope to the public, who witnessed the efforts of the government and medical institutions. Consequently, discussions on this topic increased gradually, eventually forming rumors related to epidemic control. These rumors often contained affirmations and support for regional epidemic prevention efforts, playing a significant role in boosting public confidence. In this scenario, individuals shared such rumors with others, thereby reinforcing the positive sentiments contained in the rumor content.

## 5 Discussion

### 5.1 Main findings

Based on the above research, we have identified three main findings. Firstly, at different stages of the COVID-19 pandemic, there are variations in the thematic intensity of online rumors. Specifically, during the outbreak period, rumors related to virus spread garnered widespread attention from the public. Netizens were keen on discussing the origin, transmission methods, and scope of the virus, consistent with the findings of Xiao et al. (5) and Xiong et al. (41) faced with the sudden emergence of a new virus, people were eager to learn more about the virus transmission to take appropriate protective measures (42). In subsequent stages, epidemic control and panic-inducing themes dominated online rumors, with the public consistently concerned about regional outbreaks, confirmed cases, and regional lockdown measures (43). With a more comprehensive understanding of the virus, the public subsequently focused more on the implementation of regional containment measures and the overall trajectory of the epidemic to assess future situations and take corresponding actions (44). Rumors related to production and livelihood maintained a low level of dissemination. The proportion of rumors concerning social figures remained consistently low, as the public was more interested in professionals who made outstanding contributions during the pandemic (45), a topic with naturally lower intensity.

Secondly, the emotions conveyed in the texts of online rumors undergo changes as the pandemic evolves. The proportion of rumors expressing positive emotions shows a trend of initially increasing and then decreasing. In the early stages of the pandemic, the public held optimistic views on the spread and impact of the virus (46). Positive emotions stemmed from an insufficient understanding of the epidemic and confidence in the government and medical institutions’ response measures (47, 48). As the pandemic continued to develop, the public gradually realized the severity and persistence of the epidemic, as well as the complexity and challenges of epidemic control efforts. With the daily increase in confirmed cases and the continuous strengthening of epidemic control measures, public sentiment gradually shifted toward negativity. This also explains why the proportion of negative emotions first decreases and then increases, as netizens became aware of the seriousness of the event, and emotions such as anxiety, depression, and unease spread quietly (49). In such circumstances, rumors conveying negative emotions are more likely to resonate with the public and spread (50), as they align with the current emotional state of the public, providing an outlet for expressing and appealing to reality. Rumors conveying neutral emotions are scarce at all stages, indirectly confirming the significant role played by various emotions in the dissemination of online rumors (28).

Additionally, we compared our analysis of emotional content in textual information with existing studies and found variations in emotional proportions across different types of information on various platforms. On Weibo, information related to travel safety tends to contain a higher proportion of positive emotions, whereas the term “epidemic” appears frequently in negative contexts (51). On Twitter, tweets about occupational safety with negative sentiments tend to be more popular (52), mirroring the characteristics of rumors during the pandemic. Rumors imbued with negative emotions often spread more easily. On YouTube, most of the information related to construction safety knowledge carries a predominantly positive emotional tone, while negative emotions can be effectively utilized by opinion leaders for knowledge sharing (53). This indirectly suggests that negative emotions indeed facilitate information dissemination to some extent, and during public health emergencies, rumors with negative tendencies tend to proliferate more extensively.

Finally, during public health emergencies, online rumors exhibit a co-evolution of themes and emotions. Throughout the entire event lifecycle, panic-inducing rumors intertwine with negative emotions, constantly surfacing information regarding virus mutations, transmission rates, and post-infection sequelae. For example, there have been rumors suggesting that “mass COVID-19 vaccination will accelerate virus mutation, rendering the vaccine ineffective.” However, the purpose of mass vaccination is to promptly interrupt transmission cycles and reduce mutation risks; vaccines do not accelerate virus mutations but instead decrease the likelihood of mutations. There have also been rumors claiming that “pollen can accelerate the spread of the novel coronavirus,” yet there have been no documented cases of infection caused by pollen to date. Furthermore, there are rumors asserting that “Zhong Nanshan emphasized that COVID-19 survivors will experience severe post-infection sequelae, worse than SARS,” although Zhong Nanshan himself has not made such statements; in fact, he believes that COVID-19 can be completely cured. These rumors often carry negative emotions such as fear and anxiety, triggering public concerns and panic (54). The dissemination of negative emotions further fuels the spread of panic-inducing rumors, exacerbating public anxiety and doubts. Simultaneously, the widespread dissemination of panic-inducing rumors deepens public negative emotions, creating a vicious cycle. Negative emotions and panic-inducing rumors together contribute to a negative public opinion atmosphere, posing a potential threat to public mental health and social stability (6). Epidemic control rumors often express the public’s affirmation and support for epidemic prevention measures. By disseminating positive information and attitudes, these rumors further reinforce public positive emotions, enhancing their trust and participation in epidemic prevention and control efforts (55). Meanwhile, public positive emotions also promote the spread of epidemic control rumors, accelerating the dissemination speed and scope of these rumors on social networks and media platforms. Positive emotions and epidemic control rumors together create a positive public opinion atmosphere, playing a positive role in boosting public confidence in epidemic prevention and fostering cohesion (56). Production and daily life rumors, along with virus spread rumors, interact with negative emotions during the continuous, recovery, and rebound phases, creating a negative public opinion environment, although not as significantly as panic-inducing rumors. Rumors concerning social figures maintain a low level of intensity, similar to the fluctuation range of neutral emotions. Rumors under this theme lack sufficient appeal and emotional guidance, thus failing to pique public interest.

In conclusion, we believe that during the pandemic, the interaction between the themes and emotions of online rumors mutually influences each other. On one hand, the emotions contained in rumors affect the public’s focal points, making it easier for certain types of rumors to emerge and become hot topics. On the other hand, with the emergence of rumors related to specific themes, public emotions resonate with the emotions contained in the themes, making it easier for the public to believe and spread these rumors. Consequently, the emotions contained in rumors are further amplified.

### 5.2 Implication of the research

In terms of theoretical significance, our study makes the following contributions. Firstly, we integrate the dimensions of theme and emotion in analyzing online rumors comprehensively. This analytical approach allows for a more comprehensive understanding of the dissemination mechanism of online rumors and the dynamics of public psychology. Previous studies have been limited to exploring the dissemination patterns of online rumors from either the perspective of emotion (28) or theme (57) alone, and separating the two does not yield deeper insights.

Secondly, some studies have used questionnaire surveys or laboratory experiments to investigate the reasons for the spread of rumors by the public during the COVID-19 pandemic (58, 59). However, relying solely on cross-sectional and self-reported data sources has limitations, including subjectivity and limited generalizability due to sample bias. This paper conducts research by mining the textual content of online rumors and utilizing objective data, effectively avoiding the aforementioned shortcomings.

Lastly, our study reveals the relationship between the themes and emotions of online rumors. Previous research has not clearly analyzed the connection between these two factors. We found that positive emotions are typically found in online rumors related to epidemic prevention and control, while negative emotions are more prevalent in rumors related to panic induction and virus spread. The themes’ popularity and emotional expression of online rumors mutually influence and promote each other, forming a synergistic evolution pattern of theme-emotion, elucidating the intrinsic mechanism of online rumor dissemination.

From a practical perspective, our study can offer insights and inspirations for tackling online rumors during public health emergencies. Firstly, tailored governance of online rumors at different stages of the epidemic is recommended (5). During the outbreak period, it is advisable for governments to collaborate with professional medical institutions to disseminate authoritative information objectively, addressing public concerns effectively, promptly debunking existing rumors, and guiding the public to form accurate perceptions of the epidemic. In the subsequent stages, particular attention should be paid to rumors inducing panic and those related to epidemic prevention and control. Social media platforms should strengthen information verification and management, promptly removing false information. At the same time, attention should be paid to guiding public opinion and establishing a sound intervention mechanism (60) to shield the public from the influence of rumors. During the stable period, it is crucial to promptly summarize and review the dissemination characteristics and patterns of online rumors during the event, in order to better prepare for future similar events and enhance crisis communication readiness.

Secondly, differential governance of online rumors based on different themes and emotions is essential (6, 61). Rumors inducing panic often contain negative emotions, so fostering a positive and optimistic public opinion atmosphere, such as promoting anti-epidemic deeds and regional prevention and control experiences, can counteract panic emotions and boost public confidence. Epidemic prevention and control-related rumors can leverage their positive effects by disseminating authoritative prevention and control information across various platforms, enabling the public to fully understand the actual situation in various regions and take timely personal protective measures. As for rumors related to virus spread, professional medical institutions should promptly release scientific information and expert interpretations (62, 63), debunking related rumors, while also cracking down on rumor fabrication and dissemination according to the law. Rumors related to daily life often lag behind reality, so close monitoring of public opinion dynamics is necessary to prevent phenomena disrupting social order, such as hoarding and panic buying (64, 65). Rumors related to public figures have a relatively minor impact, but objective interpretations of the words and deeds of important figures are still necessary to eliminate the negative impact of false statements.

Furthermore, improving public media literacy should be implemented as a routine task. Relevant institutions can conduct extensive media literacy promotion activities to educate the public on the importance of media literacy and methods to cultivate it (66), enhancing their understanding and identification capabilities of media information. Strengthening supervision of online platforms, regulating online information dissemination behavior, and reducing the spread of false information and rumors should also be considered. Integration of resources from universities, research institutes, and other parties to establish authoritative and professional epidemic popularization platforms on a regular basis, providing sustainable popular science services to the public, is another feasible approach.

Lastly, implementing fact-checking efforts (67) is crucial. Social media platforms can implement simple accuracy prompts to remind users to pay attention to the reliability of content before sharing it. By shifting people’s attention to accuracy, the sharing of rumors can be reduced.

### 5.3 Research limitations

Although our study provides valuable insights, it is essential to acknowledge its inherent limitations and guide future research directions. While we integrated rumor data from three platforms, there still exists a possibility of incomplete information retrieval. Rumors related to the COVID-19 pandemic are still circulating, but our dataset may not fully cover this time period, leading to missing information. Additionally, our study was conducted in China, and the situations in other countries were not considered, so the results obtained may vary due to cultural differences. Furthermore, our analysis focused on the emotions conveyed in rumor texts, which may differ from the emotions perceived by individual netizens. Lastly, our classification of emotions in online rumors was relatively coarse, dividing them into positive, negative, and neutral categories, without delving into more nuanced emotional granularity.

### 5.4 Future research directions

Future research can expand in three directions. Firstly, there is a need to deepen the dimension of emotion analysis. The current study employed relatively coarse emotion classification, and future research could introduce more nuanced emotion types, and even explore AI-based emotion analysis models. This would enable multidimensional and continuous analysis of emotions embedded in online rumor texts, providing a more accurate understanding of the trends and patterns of rumor emotions.

Secondly, conducting cross-national comparative studies is warranted. Given that the current research focuses solely on China, different countries and regions exhibit variations in cultural backgrounds, media ecologies, and other factors. These differences may lead to varied patterns and rules of rumor dissemination. Future studies could collect and compare rumor data from different countries and regions to explore the influence of cultural factors on rumor propagation, thus obtaining more universally applicable research conclusions.

Lastly, integrating new research perspectives is crucial. In addition to the thematic and emotional perspectives, future research could explore incorporating additional perspectives such as the attributes of rumor propagators, dissemination channels, and motivations. This would enrich and enhance the research findings, providing a more comprehensive understanding of online rumor dynamics.

## 6 Conclusion

During public health emergencies, online rumors reflect both public concerns and emotional tendencies. We collected rumors related to the COVID-19 pandemic from various platforms and studied the thematic shifts and emotional evolution of these rumors, analyzing the mechanisms of their co-evolution. In terms of themes, online rumors can be categorized into five main themes: epidemic prevention and control, panic-inducing, production and livelihood, virus dissemination, and social figures. These themes exhibit significant variations in popularity across different stages of the pandemic. Regarding emotions, the sentiments conveyed by online rumors change with the development of the pandemic. Rumors containing positive emotions show a trend of initially increasing and then decreasing proportions, while those containing negative emotions exhibit the opposite trend. In terms of the co-evolution of themes and emotions, rumors related to epidemic prevention and control evolve together with positive emotions, creating a positive public opinion atmosphere. Panic-inducing rumors co-evolve with negative emotions, forming a negative public opinion atmosphere. The co-evolutionary effects of rumors and emotions are not significant for the other three themes. Future research could focus on refining emotion categorization and incorporating new perspectives to better address online rumors during the next public health emergency.

## Data availability statement

The raw data supporting the conclusions of this article will be made available by the authors, without undue reservation.

## Author contributions

CS: Writing – review & editing, Conceptualization, Methodology, Project administration, Funding acquisition. ZS: Writing – original draft, Writing – review & editing, Data curation, Visualization, Funding acquisition. PH: Writing – original draft, Data curation. LL: Writing – original draft, Validation. ZX: Writing – review & editing, Data curation.

## Footnotes

^1^The URL for the China Internet Joint Rumor Refutation Platform is: https://www.piyao.org.cn/.

^2^The URL for the Rumor Filter is: https://weixin.qq.com/cgi-bin/readtemplate?t=weixin_rumor_filter.

^3^The URL for the Tencent Fact Check is: https://vp.fact.qq.com/home.
